# Prohibition on Changing Workplaces and Fatal Occupational Injuries among Chinese Migrant Workers in South Korea

**DOI:** 10.3390/ijerph16183333

**Published:** 2019-09-10

**Authors:** Ju-Yeun Lee, Sung-il Cho

**Affiliations:** 1 The Department of Public Health Science, Graduate School of Public Health, Seoul National University, Seoul 08826, Korea; inezkorea@snu.ac.kr; 2 Institute of Health and Environment, Seoul National University, Seoul 08826, Korea

**Keywords:** migrant worker, risk factor, workplace change, occupational injury, fatality, occupational safety and health

## Abstract

We assessed the risk of fatal occupational injuries among migrant workers with two different types of employment permits in South Korea. This observational study used national data from January 2007 to September 2018 and analyzed 42,089 cases of occupationally injured migrant workers of Chinese nationality. Fatality rates were analyzed according to year, sex, age, occupation, industry, and type of employment permit. Chinese workers were permitted to work for one employer and prohibited from changing employers, whereas Korean-Chinese workers were permitted to change their employer. The adjusted fatality rate of occupational injuries of Chinese migrant workers was significantly higher (1.80-fold, 95% confidence interval 1.31–2.46) than that of Korean-Chinese migrant workers. The prohibition on changing workplaces; male sex; age ≥ 45 years; machine operator; construction industry; and agriculture, livestock, and fisheries industry were risk factors for fatal occupational injuries. The results imply a need for revision of the migrant-worker employment permit systems and implementation of occupational safety and health policies for all workers to promote health equity.

## 1. Introduction

Worldwide, 2.3 million workers die annually due to occupational injuries and diseases [1]. Migrant workers have greater adverse occupational exposure and worse working conditions than native workers, resulting in adverse health outcomes such as fatal occupational injuries [2]. In 2013, the number of migrant workers reached 150.3 million globally [3], and labor migration between countries and continents is now a permanent feature of the global economy [4].

Although the occupational safety and health (OSH) of migrant workers is a global issue, only a few studies have investigated the related risk factors. While not specialized for migrant workers’ OSH, a “conceptual model for integrated approaches to protection and promotion of worker health” [5] helps understand risk factors in workplaces. The model shows that workplace policies, programs, and practices may concurrently affect the conditions of work through many pathways, e.g., the physical work environment, job tasks and demands. Conditions of work are affected by policies that determine health and safety outcomes. Policies are also emphasized in the “conceptual framework of OSH vulnerability” and there are strong associations among OSH vulnerability, policy, and the prevalence of workplace injury [6]. According to the framework, OSH vulnerability results from exposure to hazards in the workplace and inadequate mitigation resources. Migrant workers are generally included in this “vulnerable” category and in South Korea they are no exception to this. In addition, there is an unusual policy-prohibition on migrant workers changing workplaces in South Korea. Migrant workers experience higher levels of policy and procedure vulnerability [7] although policies protecting migrants’ rights may result in lower injury rates [8]. In this regard, it is necessary to find out whether the policy of prohibition on changing workplaces affects the OSH of migrant workers, however, no related research has been conducted so far.

South Korea is one of the major destination countries for migrant workers in Asia. Two kinds of permits are available under the Employment Permit System (EPS), a dependent employment permit and a work permit, although the latter are not common in South Korea. Migrant workers in other major destination countries usually work with a work permit. The policy of prohibiting a change of workplace is a distinguishing factor of working conditions for migrant workers who hold a dependent employment permit. Such migrant workers may not work for another employer unless the current employer breaks the law or the business closes [9]. The prohibition may make fatal occupational injuries more frequent because migrant workers are forced to either accept dangerous workplaces or change jobs without permission to avoid the danger. One survey in South Korea showed that poor working conditions are the main reason that migrant workers want to move to other workplaces [10]. The EPS instituted a government-to-government labor recruitment program to reduce the cost of migration, the majority of which is funded by debt [11], a major cause of overwork among migrant workers [12]. Despite the reduction in the cost of migration, the EPS has not enhanced the OSH of migrant workers. The rate of occupational injuries and disease among migrant workers increased from 0.96% in 2007 (when the EPS was initiated) to 1.08% in 2014, while the rate among native workers decreased from 0.71% to 0.51% during the same period [13,14].

The two migrant-worker employment permit systems can be evaluated by comparing groups of migrant workers of Chinese nationality with similar migration histories. The work permit system only applies to workers of Korean descent and Korean Chinese people account for 95.7% of all workers of Korean descent [15]. In September 2018, about 41.1% (*n* = 215,665) of documented migrant workers were of Chinese nationality [13]. Korean-Chinese and Chinese migrant workers share a language and culture because they are of the same nationality. Korean-Chinese people are the descendants of those who migrated to China after the 19th century due to famine, natural disasters, and the conquest of Korea by Japan, but did not return when the Communist Party of China came to power in 1949 and severed diplomatic relations with South Korea [16]. In the late 1980s, when South Korea was in the midst of rapid economic development, Korean-Chinese migrants entered the country to visit relatives and began to sell Chinese goods. In the early 1990s, the South Korean government began to allow the entry of migrant workers of other nationalities. Migrant workers can be hired only by small and medium-sized companies that are unable to recruit suitable native workers.

Korean-Chinese and Chinese migrant workers may also differ in terms of their ability to change jobs, Korean language proficiency, and whether they have relatives in South Korea. These factors may impact the fatality rate of occupational injury (FROI). Also, previous studies cite a lack of labor rights, restricted access to family and other support systems [4], and the xenophobic political climate of the host country [2] as risk factors for adverse occupational exposure and working conditions for migrant workers.

In this study, we determined whether the FROI differed due to prohibition on changing workplaces between Korean-Chinese and Chinese migrant workers. In addition, we identified the risk factors for fatal occupational injuries among Chinese migrant workers in South Korea. This is the first study of the effects of the prohibition on changing workplaces on the FROI of migrant workers.

## 2. Materials and Methods

### 2.1. Data

We used data from the Korea Workers Compensation and Welfare Services (KCOMWEL). The KCOMWEL is a national agency that manages all claims for compensation due to occupational injuries and diseases. Raw de-identified data were obtained from the KCOMWEL website through an information request system [17]. The data title is “Status of Occupational Injuries and Diseases of Migrant Workers”. The data are comprised of migrant workers’ claims for compensation from January 2007 to September 2018. The study was approved by the institutional review board (IRB) of Seoul National University.

**Figure 1:**
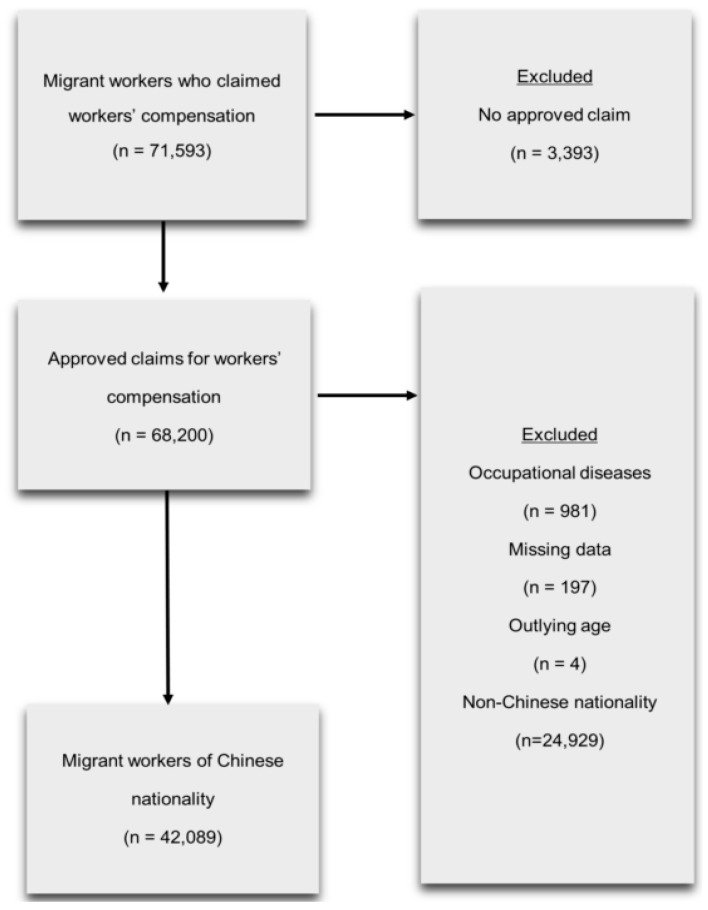
Selection of the study population.

The annual data comprised work-related injuries and diseases with fatal and non-fatal outcomes, causes and sites of injuries, demographic variables (e.g., sex, age, and nationality), and employment variables (e.g., occupation and industry). According to the KCOMWEL definitions, occupational injuries were defined as those that occurred while workers were engaged in work-related activities, including commuting (after 2018), that required >4 days of medical treatment. From 2007 to 2018, there were a total of 71,593 occupational injury or disease compensation claims, of which 42,089 were filed by migrant workers of Chinese nationality. The exclusion criteria were as conservative as possible to ensure generation of reliable results. Selection of the study population is shown in Figure 1.

The study population comprised migrant workers of Chinese nationality whose workers’ compensation claims due to occupational injuries were approved. We defined migrant workers as persons of non-Korean nationality working under the EPS irrespective of their legal status. The Ministry of Employment and Labor of South Korea has, as of September 2018, issued memoranda of understanding on migrant workers with the governments of China, Vietnam, Sri Lanka, Uzbekistan, Indonesia, Thailand, the Philippines, Nepal, Mongolia, Pakistan, Bangladesh, Cambodia, Myanmar, Kyrgyzstan, East Timor, and Laos [18]. The population of South Korea includes 2.3 million immigrants (>4%), of whom 525,000 are documented workers under the EPS. These workers originate from China (41.1%, 215,665 workers), Cambodia (7.5%, 39,122), Vietnam (7.3%, 38,075), and Nepal (6.5%, 33,906) at the end of September 2018 [13]. As of the second half of 2018, 522,595 migrant workers were employed by companies that were members of the Korea Workers Compensation Insurance, which covers 67,138 workplaces [19].

### 2.2. Measures

This observational study evaluated risk factors for fatal occupational injuries by comparing the FROIs of two groups of migrant workers of Chinese nationality. The FROI is defined as the proportion of occupational deaths among migrant workers as a result of occupational injuries. We used FROI as the outcome variable because the death count was the most reliable measure due to the high rates of under-reporting, particularly by migrant workers in South Korea. According to the OSH Research Institute of South Korea, over 70% of migrant workers who suffered an occupational injury failed to file claims for workers’ compensation [20]. A poor organizational safety climate, including management values, safety communication, safety training, and safety systems, leads to a high rate of under-reporting [21]. We thus assumed that the incidence of occupational injuries among migrant workers in South Korea had been underestimated. The difference in legal status, language, and culture, along with the high rate of under-reporting migrant workers makes it difficult to directly compare FROIs of migrant and native workers. The FROI of Korean-Chinese migrant workers was used as the reference. The workers were 15–104 years of age. Four subjects aged over 90 years old were considered outliers and removed from the analysis. The remaining workers aged from 15 to 81 years were categorized into quintiles.

Korean-Chinese migrant workers have the right to employment of their choice under the working-visit system of the EPS. To assess the effect of the prohibition on changing workplaces, we compared the FROIs of Korean-Chinese and Chinese migrant workers with adjustment for year, sex, age, occupation, industry, and site of injury. Since the start of the EPS, there have been two revisions to the law regarding the prohibition on migrant workers changing workplaces, one on 9 October 2009 and the other on 1 February 2012. Migrant workers with a dependent employment permit may apply for a workplace change only for special reasons. The 2009 revision slightly expanded the “special reasons,” and the 2012 revision clarified these reasons by providing examples; the restriction on changing workplaces remained in place. We divided the study period into three parts by the years in which revisions were issued to also assess the longitudinal effect of the related policies.

The employment of migrants is tightly controlled by the EPS with regard to both the annual numbers and the types of positions. The occupations classified by the seventh Korea Classification of Occupational Standards were divided into the four employment categories in which migrant workers were most frequently employed: manager and service workers; agriculture, livestock, and fisheries workers; machine operators; and elementary workers. Managers and service workers were grouped together due to the low fatality rates [22] in these categories. Considering that only non-professional migrant labor is permissible by the EPS, we interpreted “manager” as indicating a worker who has worked a little longer than other workers in their field, or an independent business owner. “Machine operator” included craft and related trades workers, equipment or machine operators, and assembly workers (e.g., food processing, wood and furniture, dyeing and molding, and metal-casting workers).

Nine industries were classified into five categories in which migrant workers could be employed: manufacturing, construction, service, agriculture, livestock, and fisheries. However, because there were few claims for workers’ compensation from the agriculture, livestock, and fisheries industries, we treated these as a single unit-agriculture, livestock, and fisheries (ALF). Furthermore, the transportation and delivery industries were classified as other, and the sales, food, and accommodation sectors were pooled as the service industry, in which Korean-Chinese workers account for 93.3% of the migrant workers [23]. The site of injury leading to death was classified according to the International Statistical Classification of Diseases and Related Health Problems, 10th revision (ICD-10), as head and neck, extremity, trunk, and whole body (including multiple-site injuries).

### 2.3. Statistical Analysis

The data from 42,089 migrant workers of Chinese nationality were analyzed in three stages. First, a chi-squared test was used to determine whether the FROI differed significantly according to the characteristics of the study population. Second, differences in the FROIs of each covariate were compared between the Korean-Chinese and Chinese migrant workers. Third, we identified risk factors for fatal occupational injuries using three logistic regression models with adjustment for covariates. R software (version 3.5.1) (useR, St. Louis, USA) was used to conduct all statistical analyses.

## 3. Results

### 3.1. FROI by Characteristics

The FROIs of the study population are listed in Table 1. FROIs did not differ significantly between the Korean-Chinese and Chinese migrant workers according to their characteristics. Male workers had a higher mean FROI (1.89%) than female workers (0.48%). The FROIs decreased annually and increased with worker age. The FROI of machine operators was the highest (2.34%), followed by those of ALF workers (1.74%) and elementary workers (1.36%). The industry with the highest fatality rate was construction (2.74%), followed by ALF (2.23%). The service and other industries had similar fatality rates, but the former had a lower FROI. The most frequent causes of mortality were whole-body injuries (25.95%) and head and neck (7.36%) injuries. The most frequent cause of a claim for compensation was injury to the extremities. The whole-body injuries included falls from a height, systemic burns, poisoning, multiple injuries, suffocation, and drowning.

There were differences in the causes of death due to occupational injuries according to the group and industry variables (Table 2). Collision (including falling from a height, being struck by a flying object, and being pinned under a collapsed structure) was the most common cause of mortality due to occupational injury for all occupations and industries. The deaths of machine operators had various causes, among which collision was the most frequent; the rates of other causes of mortality were similar. Cuts were the second most frequent cause of death among machine operators and elementary workers, and involved hypovolemic shock caused by stabbing or amputation. More than half of the deaths of workers in the construction industry were due to collision. Suffocation caused by fire, explosion, poison, or drowning was the second most frequent cause of occupational mortality in the service industry. The rate of suffocation was higher among Chinese workers (20.27%) than among Korean-Chinese migrant workers (11.36%). Although rolling/jamming was not always fatal, it was the major cause of permanent disability of migrant workers. In some cases, the cause of mortality was not identified; these cases were classified as “other”.

### 3.2. FROIs of Korean-Chinese and Chinese Migrant Workers

The FROIs of Chinese migrant workers according to their characteristics were equal to or greater than those of the Korean-Chinese migrant workers (Table 3). The average age of the Korean-Chinese migrant workers was 48.2 years (median 49 years), compared to an average age of 43.79 years (median 44 years) for the Chinese migrant workers. The FROI of Chinese migrant workers working in ALF (2.55%) was higher than that of Korean-Chinese migrant workers doing the same work, and higher than that of Chinese migrant workers doing other jobs.

**Figure 2:**
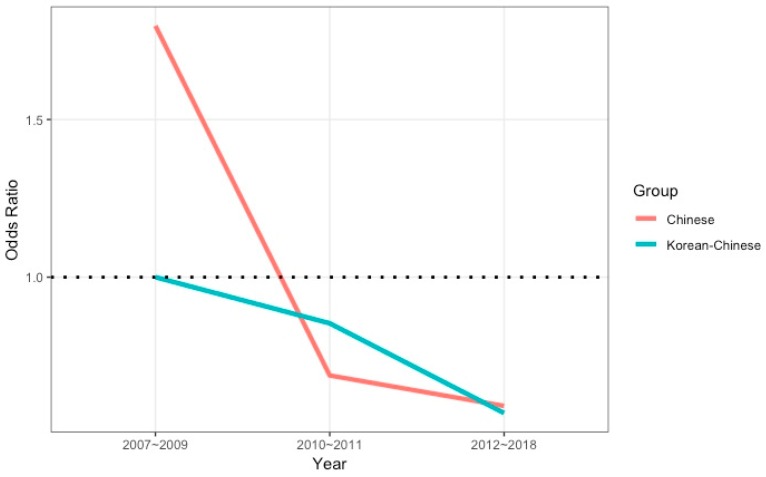
Group and year interaction odds ratio plot.

The differences in FROI between the two groups according to year allowed us to gauge the effect of the two revisions to the law regarding the prohibition on changing workplaces. Unlike Korean-Chinese migrant workers, the FROI of Chinese migrant workers did not show a decreasing tendency over time. The FROI of Chinese migrant workers decreased after 2009, when the first revision occurred, but increased after 2012, when the second revision took place. Based on the different trends in the FROI between the two groups, we evaluated the interaction effects between group and year, controlling for other variables (sex, age, occupation, and industry) (Figure 2). The interaction plot showed the odds ratios for fatalities in the two groups according to year. The odds ratios were calculated by multiplying the odds ratios from Model 3 (Table 4).

### 3.3. Factors Associated with Fatal Occupational Injuries

The FROI of Chinese migrant workers was significantly higher (1.80-fold, 95% CI 1.31–2.46 in Model 3; 1.22-fold, 95% CI 1.01–1.47 in Model 1) than that of Korean-Chinese migrant workers (Table 4) after controlling for the confounding effects of sex, age, occupation, and industry in Model 1 and for those of year and the group–year interaction in Model 3. Model 2 was controlled for year as well as for the variables in Model 1, but it did not yield a significant difference in the fatality rates of the two groups. We used the stepwise method to identify the best-fitting model and checked the goodness of fit using the Hosmer-Lemeshow test.

The FROI did not differ according to age until subjects were in their mid-40s. Compared to subjects 15–28 years of age, the FROIs of those aged 45–53 years and 54–81 years were more than two-fold higher. Thus, age ≥ 45 years was a risk factor for fatal occupational injury. Machine operators had a significantly higher (1.38-fold, 95% CI 1.00–1.88, Model 1) FROI than managers and service workers. The ORs of the construction and ALF industries were significantly higher using the service sector as the reference industry. Therefore, machine operating and the construction and ALF industries were associated with fatal occupational injuries among migrant workers.

## 4. Discussions

We evaluated factors associated with fatal occupational injuries among migrant workers in South Korea using data from 42,089 Chinese migrant workers from January 2007 to September 2018. The results showed that the prohibition on changing workplaces; male sex; age ≥45 years; machine operator; and the construction and agriculture, livestock, and fishery industries were risk factors for occupational injuries among migrant workers.

A high FROI may indicate the occurrence of occupational injuries of greater severity. Whole-body injuries were overwhelmingly fatal (Table 1). Chinese migrant workers had a higher rate of whole-body injuries and higher FROIs for the injury sites (Table 3). Alternatively, a high FROI may indicate limited access to medical services. Because the data used do not indicate legal status, identifying differences in access to medical services between the two groups was difficult. Changing workplaces without permission changes the worker’s legal status and results in forced deportation. Therefore, Chinese migrant workers are more likely to become undocumented migrants than Korean-Chinese migrant workers. With a high rate of under-reporting of occupational accidents, many migrant workers are treated under national health insurance. Undocumented migrants are not eligible for health insurance, so their access to healthcare is restricted, encouraging delay or abandonment of treatment.

The FROI of Chinese migrant workers after controlling for the potential confounding effects of covariates was 1.80-fold higher in Model 3 and 1.22-fold higher in Model 1 than that of Korean-Chinese workers. The difference in FROIs between the two groups was tied to our second research question, namely, identifying the risk factors of occupational fatal injury. The following parameters need to be considered when comparing the two groups: (1) Korean language proficiency, (2) the presence of family and relatives in South Korea, and (3) the prohibition on changing workplaces.

First, the language barrier is not an important issue in South Korea because all migrant workers, including Korean-Chinese migrant workers from EPS countries, are required to score well on the official Employment Permit System Test of Proficiency in Korean before beginning employment. Although Korean-Chinese workers have better proficiency in the Korean language than do Chinese migrant workers, Chinese migrant workers achieve higher scores than EPS workers from other regions [24], which prevents any conclusion on the impact of fluency in the Korean language on the incidence of fatal occupational injuries. Moreover, prior studies on language barriers as a risk factor for occupational injury have reported inconsistent results [25,26]. Several studies from the US and Gulf Cooperation Council countries found that a language barrier was a risk factor [2,27]. In contrast, a study in Lebanon showed that 80% of fatally injured non-Lebanese workers spoke Arabic, the native language of that country [26]. A study in South Korea suggested that lack of communication, but not language proficiency, was a risk factor as 80% of migrant workers were Korean-Chinese and were bilingual [28]. The risk related to a language barrier varies depending on the recruiting system for migrant workers in the host country. A lack of communication, e.g., no or inaccurate work instructions, rather than Korean language proficiency is more likely to be associated with fatal occupational injuries in South Korea.

Second, the right to family reunification for migrant workers in South Korea is restricted. Until 2007, Korean-Chinese migrants could obtain a labor visa only if they were invited by relatives residing in South Korea; since then, they can be granted a visa without an invitation. However, such visas do not allow migrant workers to bring their families with them. It is important to note that access to family support is not only a basic human right but also an important protective factor from occupational injury [4]. The Korean-Chinese migrant workers were not in a better position than the Chinese workers in this regard.

Therefore, the prohibition on changing workplaces, rather than language proficiency and access to family, likely explains the disparity in the incidence of fatal occupational injuries between Korean-Chinese and Chinese migrant workers. Additionally, the interaction effect between group and year (Figure 2) supports this conclusion because only the policy changed during the duration of this study. The EPS, which deprives workers of a free choice of employment had a greater deleterious impact on the fatality rate of occupational injuries to migrant workers than did the work permit system. South Korea has adopted a no-settlement principle for migrant workers, which includes a prohibition on changing workplaces. This could jeopardize the rights of migrant workers and impact their occupational health [2]. Limiting the freedom to leave employment means that workers are thoroughly subordinate to their employers. Prohibition on changing workplaces may also be used as a proxy for employers to force migrant workers to do dangerous work. Migrant workers who leave their jobs without permission are subject to deportation or become undocumented. This situation results in increased risk and severity of occupational injuries and hampers access to medical services. The prohibition on changing workplaces has been criticized, and its abolition has been recommended by the UN Committee on the Elimination of Racial Discrimination (CERD) [29], the committee of experts of the International Labor Organization (ILO) [30], and the UN General Assembly Human Rights Council [31] for promoting forced labor and human trafficking. Despite this international criticism, the provision remains in place.

The prohibition on changing workplaces has a negative impact on the health of migrant workers, and its maintenance lacks a scientific basis. According to the precedent statement and the International Organization for Migration (IOM) report, the provision exists for economic reasons. The EPS was instituted to address manpower shortages at small and medium-sized enterprises, and the South Korean government accepted employers’ claims that they would suffer from wage hikes [10]. However, on average, Korean-Chinese migrant workers change workplaces 1.27 times during their stay in South Korea according to the 2015 policy report of the IOM Immigration Policy Research Institute, a UN migration agency established based on a special relationship with the South Korean government. Migrant workers tend to change workplaces to overcome complex issues such as the labor environment, labor intensity, or human relations, rather than to obtain an increased wage, unless there is a prohibition in place [10]. Financial status is not a major consideration when migrant workers decide to change workplaces. Therefore, abolition of the provision may not disrupt the labor supply or lead to continuous wage increases.

Expanding the scope of the law regarding changing workplaces could affect the FROI of migrant workers (Figure 2). Permission to change workplaces was limited by the law to cancellation or termination of a work contract, temporary or complete closure of a business, cancellation or restriction of employment permits, and injury of workers; all are related to employers. The possibility of changing workplaces was increased slightly by the addition of “violation of working conditions, unfair treatment” to the law at the end of 2009; indeed, this resulted in a rapid decrease in the FROI of Chinese migrant workers compared to that of Korean-Chinese migrant workers. In contrast, the revision in early 2012 only clarified the scope of the law, but the subsequent increase in the FROI of Chinese migrant workers suggests that expanding the possibility of changing workplaces was of limited utility in terms of reducing the fatality rate of workers.

Injured male migrant workers were more likely to die than were injured female migrant workers. However, in South Korea, only workers who meet the criteria of the Labor Standards Law can claim workers’ compensation under the Korea Workers’ Compensation Insurance Act. It is thus possible that some female migrant workers were unable to claim workers’ compensation because they were not legally defined as workers. In the case of domestic workers who perform domestic duties such as cleaning, cooking, and looking after children or elderly people [9], the overwhelming majority were female and 92.8% were classified as self-employed; i.e., not legal workers [32]. Being male is reportedly a risk factor for migrant workers, but there are marked disparities in mental-health outcomes, cancer rates, occupational injuries, and reproductive-health outcomes between male and female workers [2]. Females comprise only 8.8% of non-professional migrant workers in South Korea, but 40.4% of all migrant workers of Korean descent [13]. The different employment patterns of female and male migrant workers may mean that they face different occupational hazards.

Age ≥45 years was a risk factor for fatal occupational injuries among migrant workers, as reported previously [4,25]. In contrast, older native workers in the US had a low injury rate. This was attributed to the healthy worker effect whereby older workers retire from high-risk jobs or move to less demanding jobs [33,34]. However, no such effect was identified for older migrant workers who may need to remain in high-risk occupations [25,35].

Machine operation was the highest-risk occupation, and construction and ALF were the most physically demanding industries. Only machine operators had a significantly high risk of occupational injury, compared to managers and service workers. Construction and ALF were risk factors for fatal occupational injuries, consistent with previous reports [36,37]. The ALF industry had a high FROI but few (*n* = 26) fatalities, suggesting that ALF workers could not claim workers’ compensation unless they were severely injured.

This study has several limitations. First, persons not defined as workers according to South Korean law are omitted from the workers’ compensation claims data; thus, further studies that include such workers are needed. Second, the occupational categories may have been inaccurate. The colleagues or agents who reported deaths may have misclassified the occupation of the deceased [38]. Despite these limitations, we used FROI as the dependent variable because data on workers’ compensation for fatal occupational injuries provide reliable information about occupational deaths [39,40], and because mortality data are the most reliable measure when under-reporting rates are high.

A longitudinal follow-up study of the health of migrant workers is needed to ascertain whether the prohibition on changing workplaces affects not only occupational injuries but also occupational diseases. Because this was basically an observational cross-sectional study, we could not control for missing or unmeasured factors, and were unable to establish causality. The importance of this study lies in its comparison of recruitment systems and it being the first investigation of the effect of the prohibition on migrant workers changing workplaces on fatal occupational injuries.

## 5. Conclusions

We compared two groups of migrant workers of Chinese nationality with different types of employment permits under the EPS. Of the two types of permits, it was found that the FROI for the group with the dependent employment permit, which prohibits changing workplaces was significantly higher. The status of occupational injuries and diseases of migrant workers’ data revealed the prohibition on changing workplaces to be a risk factor for fatal occupational injury. Additionally, male sex; age ≥45 years; machine operator; construction; and the agriculture, livestock, and fishery industries were associated with fatal occupational injuries among migrant workers in South Korea. Policies to improve the recruiting system and to strengthen workplace safety measures for better OSH of migrant workers are the responsibility of the global community as well as local governments and businesses. Ultimately, preventing occupational injuries to migrant workers in high-risk occupations will improve the health of all workers.

## Figures and Tables

**Table 1:** FROI ^a^ according to subjects’ characteristics (*n* = 42,089).

Variables	Total			FROI ^a^		
		*n*	%	*n*	%	*p*-Value ^#^
		42,089	100.00	686	1.63	
**Group**						0.97
Korean-Chinese		32,987	78.37	538	1.63	
Chinese		9102	21.63	148	1.63	
**Year**						<0.01
2007–2009		7515	17.86	174	2.32	
2010–2011		14,765	35.08	250	1.69	
2012–2018		19,809	47.06	262	1.32	
**Sex**						<0.01
Female		7875	18.71	38	0.48	
Male		34,214	81.29	648	1.89	
**Age**						<0.01
15–28		2130	5.06	16	0.75	
29–36		5134	12.20	62	1.21	
37–44		8307	19.74	110	1.32	
45–53		13,737	32.64	234	1.70	
54–81		12,781	30.37	264	2.07	
**Occupation**						<0.01
Manager & Service worker		9248	21.97	84	0.91	
ALF ^b^ worker		516	1.23	9	1.74	
Machine operator		15,665	37.22	367	2.34	
Elementary worker		16,660	39.58	226	1.36	
**Industry**						<0.01
Manufacturing		16,845	40.02	186	1.10	
Construction		13,873	32.96	380	2.74	
ALF ^b^		1166	2.77	26	2.23	
Service		6772	16.09	45	0.66	
Others		3433	8.16	49	1.43	
**Site of Injury**						<0.01
Head & Neck		4703	11.17	346	7.36	
Extremity		31,542	74.94	30	0.10	
Trunk		5031	11.95	99	1.97	
Whole body		813	1.93	211	25.95	

*Note*: **^#^** All *p*-values were from chi-squared tests. ^a^ Fatality Rate of Occupational Injury, ^b^ Agriculture, Livestock and Fisheries.

**Table 2:** Mechanisms of deaths due to occupational injuries according to subjects’ characteristics (*n* = 686).

Variables	Collision		Cuts		Suffocation		Rolling/Jamming			Others	Total		*p*-Value ^#^
	*n*	%	*n*	%	*n*	%	*n*	%	*n*	%	*n*	%	
**Group**													0.04
Korean-Chinese	278	51.77	101	18.81	61	11.36	33	6.15	65	11.92	538	78.43	
Chinese	75	50.68	23	15.54	30	20.27	9	6.08	11	7.43	148	21.57	
**Occupation**													0.06
Manager & Service worker	44	52.38	11	13.10	19	22.62	3	3.57	7	8.33	84	12.24	
ALF ^a^ worker	4	44.44	1	11.12	4	44.44	0	0.00	0	0.00	9	1.31	
Machine operator	184	50.14	66	17.98	50	13.62	25	6.81	42	11.44	367	53.50	
Elementary worker	121	53.54	46	20.35	18	7.96	14	6.19	27	11.96	226	32.94	
**Industry**													<0.01
Manufacturing	71	38.17	40	21.51	25	13.44	29	15.59	21	11.29	186	27.11	
Construction	222	58.42	67	17.63	39	10.26	6	1.58	46	12.11	380	55.39	
ALF ^a^	13	50.00	4	15.38	7	26.93	2	7.69	0	0.00	26	3.79	
Service	17	37.78	6	13.33	13	28.89	1	2.22	8	17.78	45	6.56	
Others	30	61.22	7	14.29	7	14.29	4	8.16	1	2.04	49	7.14	

^a^ Agriculture, Livestock and Fisheries. **^#^** All *p*-values were from chi-squared or Fisher’s exact tests.

**Table 3:** FROI ^a^ of Korean-Chinese and Chinese migrant workers (*n* = 42,089).

Variables	Korean-Chinese (*n* = 32,987)			Chinese (*n* = 9102)			*p*-Value ^#^
	*n*	Death	FROI ^a^ (%)	*n*	Death	FROI ^a^ (%)	
**Sex**							0.08
Female	6114	26	0.43	1761	12	0.68	
Male	26,873	512	1.91	7341	136	1.85	
**Year**							<0.01
2007–2009	5109	105	2.06	2406	69	2.87	
2010–2011	12,205	221	1.81	2560	29	1.13	
2012–2018	15,673	212	1.35	4136	50	1.20	
**Age**							<0.01
15–28	873	1	0.11	1257	15	1.19	
29–36	3653	39	1.07	1481	23	1.55	
37–44	6282	78	1.24	2025	32	1.58	
45–53	11,552	192	1.66	2185	42	1.92	
54–81	10,627	228	2.15	2154	36	1.67	
**Occupation**							<0.01
Manager & Service worker	7415	68	0.92	1833	16	0.87	
ALF ^b^ worker	359	5	1.39	157	4	2.55	
Machine operator	12,531	288	2.30	3134	79	2.52	
Elementary Worker	12,682	177	1.40	3978	49	1.23	
**Category of Business**							<0.01
Manufacturing	12,533	143	1.14	4312	43	1.00	
Construction	11,413	298	2.61	2460	82	3.33	
ALF ^b^	931	21	2.26	235	5	2.13	
Service	5334	35	0.66	1438	10	0.70	
Others	2776	41	1.48	657	8	1.22	
**Injury Site**							<0.01
Head & Neck	3720	282	7.58	983	64	6.51	
Extremity	24,493	24	0.10	7049	6	0.09	
Trunk	4148	79	1.90	883	20	2.27	
Whole body	626	153	24.44	187	58	31.02	

^a^ Fatality Rate of Occupational Injury, ^b^ Agriculture, Livestock and Fisheries, **^#^** All *p*-values were from chi-squared test.

**Table 4:** Odds ratios for fatality rate of occupational injuries between Korean-Chinese and Chinese migrant workers by models.

Variables	Model 1		Model 2		Model 3	
	OR ^a^	95% CI ^b^	OR ^a^	95% CI ^b^	OR ^a^	95% CI ^b^
**Group**						
Korean-Chinese	1.00	-	1.00	-	1.00	-
Chinese	1.22	1.01–1.47 *	1.18	0.97–1.42	1.80	1.31–2.46 ***
**Year**						
2007–2009			1.00	-	1.00	-
2010–2011			0.70	0.57–0.86 ***	0.85	0.67–1.09
2012–2018			0.48	0.39–0.58 ***	0.57	0.45–0.72 ***
**Group:Year**						
Chinese * (2007–2009)					1.00	-
Chinese * (2010–2011)					0.45	0.27–0.74 **
Chinese * (2012–2018)					0.58	0.37–0.90 *
**Sex**						
Female	1.00	-	1.00	-	1.00	-
Male	2.86	1.96–4.01 ***	2.80	1.95–4.00 ***	2.81	1.96–4.02 ***
**Age**						
15–28	1.00	-	1.00	-	1.00	-
29–36	1.49	0.87–2.63	1.49	0.85–2.59	1.46	0.84–2.54
37–44	1.52	0.90–2.61	1.46	0.86–2.50	1.45	0.85–2.47
45–53	2.06	1.20–3.40 **	2.03	1.21–3.41 **	2.01	1.19–3.37 **
54–81	2.47	1.42–3.99 **	2.54	1.51–4.26 ***	2.53	1.50–4.24 ***
**Occupation**						
Manager & Service worker	1.00	-	1.00	-	1.00	-
ALF ^c^ worker	0.75	0.34–1.65	0.78	0.35–1.73	0.79	0.36–1.75
Machine operator	1.38	1.00–1.88 *	1.35	0.99–1.85	1.37	1.00–1.87
Elementary worker	0.79	0.56–1.09	0.78	0.56–1.08	0.79	0.56–1.10
**Industry**						
Service	1.00	-	1.00	-	1.00	-
Manufacturing	1.10	0.71–1.70	1.06	0.69–1.64	1.06	0.68–1.63
Construction	2.40	1.56–3.68 ***	2.48	1.62–3.80 ***	2.46	1.61–3.76 ***
ALF ^c^	2.13	1.21–3.77 **	1.96	1.10–3.50 *	1.92	1.08–3.44 *
Others	1.34	0.84–2.14	1.26	0.78–2.02	1.24	0.77–2.00

Note: Estimates were obtained by logistic regression with logit as a binomial link function. Model 1 was adjusted for sex, age, occupation, and industry. Model 2 added year to Model 1 for confounding factors. Model 3 showed interactions between group and year variables. ^a^ OR: Odds Ratio, ^b^ CI: Confidence Interval, ^c^ Agriculture, Livestock and Fisheries, * *p* < 0.05, ** *p* < 0.01, *** *p* < 0.001.
